# Identification of novel Bach2 transcripts and protein isoforms through tagging analysis of retroviral integrations in B-cell lymphomas

**DOI:** 10.1186/1471-2199-10-2

**Published:** 2009-01-21

**Authors:** Jinghua Liu, Annette Balle Sørensen, Bruce Wang, Matthias Wabl, Anders Lade Nielsen, Finn Skou Pedersen

**Affiliations:** 1 Department of Molecular Biology, University of Aarhus, Aarhus, DK 8000, Denmark; 2 The State and University Library, Aarhus, DK 8000, Denmark; 3 Picobella, L.L.C, 863 Mitten Road, Suite 101, Burlingame, CA 94010, USA; 4 Department of Microbiology and Immunology, University of California, San Francisco, CA-94143, USA; 5 Department of Human Genetics, University of Aarhus, Aarhus, DK 8000, Denmark; 6 Department of Molecular Biology, C.F. Møllers Allé 1.130, University of Aarhus, DK-8000 Aarhus C, Denmark

## Abstract

**Background:**

The *Bach2 *gene functions as a transcriptional repressor in B-cells, showing high expression level only before the plasma cell stage. Several lines of evidence indicate that Bach2 is a B-cell specific tumor suppressor. We here address patterns of insertional mutagenesis and expression of Bach2 is a murine retroviral model of B-cell lymphoma induction.

**Results:**

We report that the *Bach2 *gene is a target of proviral integrations in B-cell lymphomas induced by murine leukemia virus. An alternative *Bach2 *promoter was identified within intron 2 and this promoter was activated in one of the tumors harboring proviral integration. The alternative promoter was active in both normal and tumor tissue and the tissue specificity of the two *Bach2 *promoters was similar. Three different alternatively used *Bach2 *terminal exons were identified to be located in intron 4. The inclusion of these exons resulted in the generation of *Bach2 *mRNA with open reading frames lacking the bZIP DNA binding domain present in the normal Bach2 protein, but retaining a partial BTB protein dimerization domain. Such Bach2 protein was excluded from the cell nucleus.

**Conclusion:**

We have identified an alternative promoter and new protein isoforms of Bach2. Our data imply that activation of an alternative promoter by proviral integration serves as a possible mechanism of up-regulation of the *Bach2 *gene with a potential role in B-cell lymphomagenesis. The finding of novel *Bach2 *transcripts and protein isoforms will facilitate a better insight into the normal and pathophysiological regulation of the *Bach2 *gene.

## Background

The transcription factor Bach2 (BTB and CNC homolog 2) is a member of the family of proteins harboring a basic region leucine zipper (bZip) DNA binding domain [1]. In addition, Bach2 possesses a BTB domain. Both of these domains are involved in forming heterologous protein-protein interactions [2-4]. In mice abundant Bach2 expression is described in neuronal cells, monocytes, and in the B-cell compartment only before the plasma cell stage [1,5,6]. The sub-cellular localization of the Bach2 protein is controlled by the cytoplasmic localization signal present in the bZip domain and a C-terminal nuclear-export signal. Bach2 is localized in the cytoplasm through its C-terminal nuclear export signal [7]. In B-cells, phosphorylation of Bach2 by the PI3/S6 kinase pathway results in the cytoplasmic accumulation of Bach2 [8]. Nuclear accumulation of Bach2 is induced by anticancer drugs with oxidative stress activities and is regulated by small ubiquitin like modifier-1 or by SUMOylation [9,10]. Bach2 nuclear foci are observed to be associated with promyelocytic leukemia nuclear bodies in apoptosis [11].

Bach2 proteins function as transcriptional repressors and form heterodimers with small Maf oncoproteins (MafF, MafG, MafK). Such heterodimers bind to the Maf recognition elements [1]. As an example, Bach2 negatively regulates the immunoglobulin heavy chain gene by binding to the Maf recognition element in the 3'-enhancer [6]. Besides, Bach2 is crucial for the programming of antibody class switching and somatic hypermutation of immunoglobulin genes [12]. Several lines of evidence show that Bach2 is a B-cell specific tumor suppressor. For example, in non Hodgkin's Lymphoma, a relatively high frequency of loss of heterozygosity was detected for Bach2 [13]. Moreover, the Bach2 expression level has proven to be a useful marker to predict disease-free and overall survival of patients with diffuse large B-cell lymphoma, where a favorable prognosis is correlated with a high expression level of Bach2 [14]. In consistence with its role as a putative tumor suppressor, Bach2 was found to induce apoptosis in response to oxidative stress [7]. Over-expression of Bach2 increased cellular toxicity of anticancer drugs that generate reactive oxygen species [9]. In the Burkitt lymphoma cell line Raji, loss of Bach2 expression at both the mRNA and protein levels was attributed to Epstein-Barr virus (EBV) genome integration into the host Bach2 gene [15]. The enforced expression of Bach2 in the Raji cell line led to a marked reduction of clonogenity [13]. Moreover, Bach2 was seen down-regulated in proliferating lymphoblastoid cell lines, which were *in vitro *transformed by EBV from resting B-cells [16]. These findings suggest that loss or down-regulation of Bach2 expression may contribute to B-cell lymphomagenesis.

Proviral insertional mutagenesis plays an important role in lymphomagenesis by non-acutely transforming murine leukemia viruses (MLVs). By insertion of proviral DNA into the host genome, the retrovirus may activate cellular proto-oncogenes, or more rarely repress tumour suppressor genes [17-19]. Thus, loci or genes repeatedly found to be targeted in retrovirus-induced tumors most likely play important roles in the disease process. The specific genes that are tagged by a provirus in a given retrovirus-induced tumor depend on virus type as well as on mouse genetic background [20,21].

In the present study we have examined tumors induced by wild-type and mutants of Akv MLV in inbred NMRI mice. In these models the tumors are of B-cell lineage with frequent occurrence of plasmacytomas/plasma cell proliferation [22-24]. Recently, we proposed that polyclonal immune stimulation and insertional mutagenesis exert dual effects in the process of disease induction in this Akv/inbred-NMRI model [23]. We here describe the *Bach2 *locus as a prominent target in MLV induced B-cell lymphogenesis. A cluster of integrations was detected in intron 2 of the *Bach2 *gene. Interestingly, in the same intron we have identified an alternative *Bach2 *promoter. We also identified alternatively used *Bach2 *terminal exons located within intron 4. Utilization of alternative promoter sequences and terminal exons resulted in new *Bach2 *mRNA subtypes, of which several have coding potential for Bach2 protein isoforms containing a partial BTB protein-protein interaction domain but lacking the bZIP DNA binding domain. Altogether, the presented data show a novel regulatory complexity resulting in the generation of different Bach2 proteins.

## Methods

### Mouse tumors and tissues

Tumors used in this study were induced in NMRI inbred mice by Akv MLV variants from previous and unpublished work [22,24,25]. In brief, infectious viral particles of Akv MLV and derivatives hereof were inoculated into newborn inbred NMRI mice. Upon diagnosis of lymphomas, the animals were sacrificed and lymphomas were dissected and frozen (criteria for diagnosis were described previously [26]). Tissues from mock-injected or untreated mice served as controls. All animal studies were in accordance with the German Animal Welfare Act. They were approved by the Institutional Animal Care and Use Committee (IACUC) of the Helmholtz Center Munich and by the ethical committee of the Government of Upper Bavaria, Germany (211-2531-48/98 and 55.2-1-54-2531-98-03).

### Genomic DNA and total RNA Isolation and Quantification

Genomic DNA and total RNA were isolated from frozen tumors and tissues using DNeasy Tissue Kit (Qiagen) and TRIzol^® ^Reagent (Invitrogen™), respectively. Quantification was performed by a spectrophotometer.

### PCR identification and verification of provirus integration sites

Genomic DNA isolated from the induced tumors was analyzed for provirus integration sites by a splinkerette-based PCR method [27] described in details elsewhere [28]. To confirm provirus integrations into the *Bach2 *gene, PCR were done on genomic DNA from tumors with a gene specific primer and a viral primer. The gene specific primers at different proviral integration sites were designed according to integration site data. Viral primers 2620 or 6197 were both described previously [22,29]. The PCR was run in a 25-μl volume containing 0.625 U of *Taq *DNA polymerase (5 U/μl; Invitrogen), 1.5 mM MgCl_2 _(Invitrogen), 2.5 μl of 10× *Taq *buffer (Invitrogen), 0.2 mM of each deoxynucleoside triphosphate (Invitrogen), and 10 pmol of each primer. The fragments were amplified in a 2720 thermal cycler (Applied Biosystems) with a touch-down program as follows: 1 cycle of denaturation at 94°C for 5 min and then 10 cycles of denaturation at 94°C for 30 s, annealing at 64–55°C for 30 s with 1 cycle decreasing 1°C, and extension at 72°C for 3 min followed by 30 cycles of denaturation at 94°C for 30 s, annealing at 55°C for 30 s, and extension at 72°C for 3 min, and finally 1 cycle of extension at 72°C for 10 min.

### Southern blot analyses

Twenty micrograms of genomic DNA from each sample was digested with *Hin*dIII or *Nco*I, separated on a 0.8% agarose gel, transferred to a Zeta-Probe membrane (Bio-Rad), and hybridized with ^32^P labelled Bach2 specific DNA probes or an ecotropic MLV-specific envelope probe (Eco-*env*). The Bach2 gene-specific DNA probes were PCR products of 892 bp or 743 bp, amplified with the following primer pairs: probe1; 5'-TCTAGGGTTCAGGTGGGATG-3' and 5'-GCACAAGTGCTGGCTAACAA-3'; probe2; 5'-ACTTCAGGCTACTGCCCAGA-3' and 5'-CACATGGAGACGGTTGTGAC-3'. Probes were purified from gel bands with GFX columns. The Eco-*env *probe and detailed procedures for blotting and hybridization were described previously [22,30].

### RT-PCR, Q-PCR, and sequencing

For generation of RT-PCR templates first-strand cDNA was synthesized from 200 ng total RNA with an oligo dT primer kit (GE Healthcare) and for experiments comparing oligo dT primed and random-primed cDNA synthesis with the RevertAid H Minus First Strand cDNA Synthesis Kit (Fermentas). RT-PCR reactions were performed with the following program: 1 cycle of denaturation at 94°C for 5 min and then 40 cycles of denaturation at 94°C for 30 s, annealing at 60°C for 30 s and extension at 72°C for 3 min, and finally 1 cycle of extension at 72°C for 10 min. PCR products were separated by agarose gel electrophoresis and purified with GFX columns before subjected to sequence determination by ABI 7300 Biosystems. Quantitative real-time RT-PCR (Q-PCR) was performed on a MX4000™ Multiplex Quantitative PCR system (Stratagene) or on a lightcycler (Roche). For each reaction, first-strand cDNA from 20 ng of total RNA was used. All reactions were done in triplicates. The amplification efficiencies of *Bach2 *amplicons were calculated by the use of standard curve analysis where the Q-PCR templates were serial dilutions of purified *Bach2 *cDNA derived from spleen and tumor tissue. *Bach2 *mRNA expression levels were normalized to the expression level for tbp (TATA-box binding protein). Detailed information concerning primer sequences is available upon request.

### 5'-Rapid amplification of cDNA end (RACE) analyses

The 5' sequences of *Bach2 *isoforms were determined by 5' RACE analyses using SMART™ RACE cDNA Amplification kit (Clontech) according to the manufacturer's instructions with slight alterations. Briefly, the 5' RACE-ready cDNA was synthesized with 1 μg of total RNA from the mouse tumor ID:99–1206. The 5' sequences were then amplified with the forward universal primer mix (UPM, Clontech) and two Bach2 isofom B specific reverse primers 1e and 1f, respectively. The primer sequences were as follows: 1e, 5'GTGGCTATGATCCAGTCACCCCGATCT-3'; and 1f, 5'-ATGAGTGTTGCACACCGTGAATCTCCTG-3'. RACE PCR was performed with the following program: 1 cycle of denaturation at 94°C for 5 min and then 35 cycles of denaturation at 94°C for 30 s, annealing at 68°C for 30 s, and extension at 72°C for 3 min, and finally 1 cycle of extension at 72°C for 10 min. RACE products were sequenced by means of ABI 7300 Biosystems with primers UPM, 1e, 1f and another two nested gene-specific primers 1b, 5'-ACGCACACACACTCCACACCCTGAAAG-3', and 1c, 5'-ACACGCACACACACTCCACACCCTGAAA-3', respectively.

### Cell culture, transfection and immunofluorescence staining

NIH 3T3 murine fibroblasts and HEK 293T human embryonic kidney cell line were cultured at 37°C with 5% CO_2 _in Dulbecco's modified Eagle's medium containing Glutamax-I (Gibco) supplemented with 10% newborn calf serum or foetal bovine serum, respectively, and with 100 U/ml penicillin and 100 μg/ml streptomycin. Transfections of NIH 3T3 cells were performed using Lipofectamine Reagent (Invitrogen) following the manufacturer's protocol. HEK 293 T cells were transfected by the calcium phosphate precipitation method [31] using 0.5 μg/cm^2 ^DNA. Forty-eight hours after transfection, cells were fixed by para-formaldehyde, immunostained with anti-FLAG antibody and with TRITC-conjugated secondary antibody, mounted with DAPI-mounting solution (Invitrogen), and subjected to fluorescence monitoring by epi-fluorescence microscopy.

### Protein extraction and Western Blot analyses

Protein samples were extracted from frozen tumors or cultured cells 48 h post-transfection with lysis buffer (50 mM Tris-HCl (pH 8.0), 150 mM NaCl, 1% NP-40, 0.5% sodium deoxycholate, 0.1% SDS and 1 mM PMSF). Samples containing 10 μg total protein (BCA™ Protein Assay Kit, Pierce Biotechnology) were resolved on a 12.5% SDS-PAGE gel and electro-transferred onto a polyvinylidene fluoride (PVDF) membrane (Millipore Corporation). The membrane was blocked in TBS (20 mM Tris-HCl, 200 mM NaCl, pH7.6) containing 0.05% Tween-20 (TBS-T) and 5% (w/v) fat-free milk. The blot was hybridized for 1 h with goat anti-mouse Bach2 polyclonal antibody against the N-terminus of the Bach2 protein (sc-14702) (Santa Cruz Biotechnology) in dilution of 1:1000 in TBS-T containing 5% fat free milk. The blot was washed twice in TBS-T and then incubated with secondary antibody of horseradish peroxidase (HRP)-conjugated rabbit anti-goat immunoglobulins/HRP (DAKO) with 1:5000 dilutions in TBS-T containing 5% fat-free milk. The membrane was washed twice in TBS and subjected to Bach2 protein detection by ECL Plus Western Blotting Detection System (GE Healthcare) before being exposed to a medical film (Konica Minolta Medical and Graphic Inc.). The membrane was stripped and re-hybridized with 1:5000 dilutions of goat polyclonal anti-human Beta-Actin antibody (sc-1616) (Santa Cruz Biotechnology) for protein loading control. Protein samples extracted from transfected cells were analyzed by Western blots using anti-FLAG M2 peroxidase-conjugated monoclonal antibody (Sigma) according to the recommended procedure but using a 1:5000 dilution of antibody.

## Results

### Identification of 18 provirus integrations into the Bach2 locus

**Figure 1:**
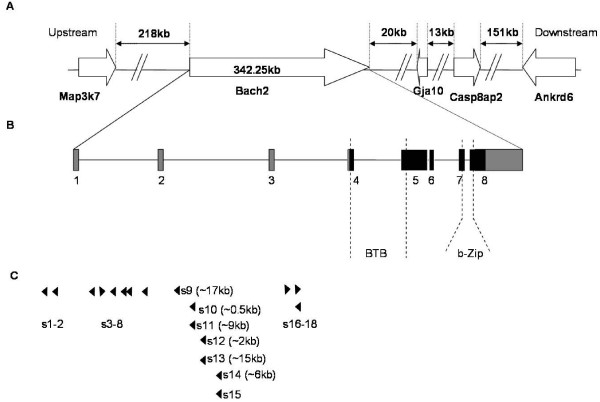
**The *Bach2 *locus and proviral integration sites**. Structures of genes, exons, and distances between insertion sites are not shown in scale. (A) Schematic description of the mouse chromosome 4 region around the *Bach2 *locus. The transcriptional orientation of the genes is indicated by arrows. (B) Illustration of the *Bach2 *gene. The exons are shown with solid bars, with un-translated regions in grey and the coding sequences in black. The dashed lines indicate the location of Bach2 protein domains, BTB and bZip. (C) Illustration of the localization of proviral insertions in the *Bach2 *gene. Position and transcriptional orientation of the proviruses are shown by triangles and were confirmed by gene-specific PCRs. The proviruses are named after the integration order from left to right at the locus, with s1 being the first integration site and s18 the last. For the proviruses located in intron 2, distances away from the nearest upstream proviral integration site are shown in brackets in base pairs.

In inbred NMRI mice, wild-type and mutants of Akv MLV induce tumors of B-cell lineage with frequent occurrence of plasmacytomas/plasma cell proliferation [22-24]. To identify cellular genes involved in the disease process provirus integration sites were mapped in dissected tumors. Out of approximately 2000 identified integration sites 18 were by PCR confirmed to map to the *Bach2 *locus (Figure 1 and Table 1). Since proviral insertion into the host genome is essentially a random event, such a frequent observation of proviruses within the *Bach2 *locus strongly supports a role in lymphomagenesis of the *Bach2 *gene. As shown in Figure 1 and Table 1, the proviruses were integrated in the non-coding region; two were located within the promoter region; six within intron 1; seven within intron 2; and three within intron 3. All but three integrated proviruses were inserted in opposite orientation relative to the transcriptional orientation of *Bach2*.

**Table 1:** Summary of proviral integration in NMRI-i mice into the *Bach2 *gene

No.	Mouse ID	Virus variant^a^	Region	Orientation^b^	PCR^c^	Southern blots^d^
S1	03–655	Akv 1–99Runx	5' promoter	-	+	-
S2	99–64	Akv PBS-Gln	5' promoter	-	+	-
S3	99–148	Akv PBS-Gln	Intron1	-	+	-
S4	01–1124	Akv 1–99mRunx+Egre	Intron1	+	+	n.d.
S5	98–1286	Akv PBS-Gln	Intron1	-	+	n.d.
S6	99-97	Akv PBS-Gln	Intron1	-	+	-
S7	01–454	Akv 1–99wt	Intron1	-	+	-
S8	98–1197	Akv PBS-Pro	Intron1	-	+	-
S9	99–955	Akv 1–99wt	Intron2	-	+	-
S10	99–1020	Akv 1–99wt	Intron2	-	+	-
S11	99–1206	Akv 1–99wt	Intron2	-	+	-
S12	03–858	Akv 1–99mGR	Intron2	-	+	-
S13	99–128	Akv PBS-Lys	Intron2	-	+	-
S14	99–128	Akv PBS-Lys	Intron2	-	+	-
S15	03–653	Akv 1–99mEgre	Intron2	-	+	-
S16	99-74	Akv PBS-Gln	Intron3	+	+	-
S17	03–290	Akv/SL3-3 TM	Intron3	+	+	n.d.
S18	99-95	Akv PBS-Arg	Intron3	-	+	-

^a^The virus variants originated from our published [22,24,25,30,56-60] or unpublished pathogenicity work. ^b^The virus was transcribed in the same (+) or opposite (-) orientation compared to that of the mouse *Bach2 *gene. ^c^Provirus was detectable (+) or undetectable (-) by PCR. ^d^Southern blots showed the proviral integration to be present in a minor fraction of cells in tumor tissue (-). N.d., not determined.

In order to clarify if the identified provirus integrations were present in a large fraction of cells in their respective tumors, we performed Southern Blot analyses on tumor genomic DNA. We did not observe any rearrangement of genomic DNA, corresponding to provirus integrations, using *Bach2 *gene specific probes (data not shown). Rehybridization with a provirus-specific ecotropic envelope probe confirmed this observation, since no hybridizing fragments were detected (data not shown). Tissues from Balb/c mice containing a single endogenous ecotropic provirus were used as positive controls (data not shown). Together this indicated that only a minor proportion of the cells contained the actual proviral integration in end-stage tumor tissue.

### Examination of the Bach2 mRNA expression level in tumors

**Figure 2:**
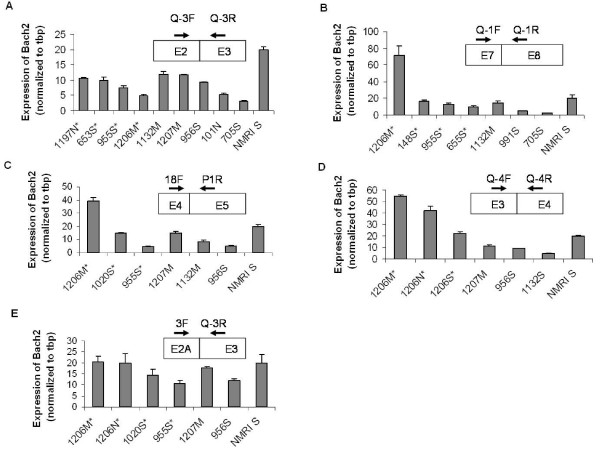
**Expression analysis screened out a *Bach2 *integration site with high mRNA expression level**. Q-PCR was performed on total RNAs from tumors with (asterisked) or without proviral integration in *Bach2*. The used primer pairs and their schematic localization is shown in (A), (B), (C), (D), and (E) (for localization and origin of exon 2A, see figure 5 and text). M, N, and S (mesenteric, cervical (neck) lymph node, and spleen, respectively) refer to types of tumor tissues used for the analyses. *Bach2 *expression levels were normalized to the tbp expression level and the *Bach2 *expression level in spleen tissue from untreated NMRI mice (NMRI S) was given the value 20.

To estimate *Bach2 *expression levels in tumors quantitative real-time RT-PCR (Q-PCR) was carried out on cDNA from tumors with provirus insertion in the Bach2 locus. As controls, tumors with no *Bach2 *locus integration were included together with normal spleen tissue. Using a primer combination spanning *Bach2 *exons 2 to 3 we observed no significant difference in the *Bach2 *expression level between the two types of tumor cohorts (Figure 2A). We notice a lower level of *Bach2 *mRNA expression in all tumor samples compared to the normal spleen control (Figure 2). Using a primer pair covering exons 7 to 8, we noticed the same tendency, except for tumor 1206, which displayed a marked increase in *Bach2 *mRNA expression, compared to that of the other tumor samples and normal spleen (Figure 2B). The discrepancy between the results obtained for the different primer combinations for this particular tumor sample led us to proceed with other primer combinations. Also for primer combinations spanning *Bach2 *exons 4 to 5 (Figure 2C) and exons 3 to 4 (Figure 2D) an up-regulation of Bach2 expression in tumor 1206 was evident as compared to the other tumor samples. Thus, in tumor 1206, a preferential up-regulation of *Bach2 *transcripts including exon sequences spanning exon 3 to exon 8 was observed. Western blot analyses were carried out with a polyclonal antibody identifying the N-terminus of the Bach2 protein encoded by exon 4. We detected an even expression level of a 110-KDa protein, corresponding to the expected size of Bach2 protein [1], in tumors from mouse 1206 and other tumors (data not shown).

### Identification of an alternative Bach2 promoter

The observation of an increased *Bach2 *mRNA expression level specifically for exon sequences located downstream of exon 2 in tumors from mouse 1206 let us hypothesize that this was due to activation of alternative promoter sequences present within intron 2 of the *Bach2 *gene. Such promoter sequences would result in the generation of a novel first exon if appropriate splice donor sequences were present or alternatively in a continuous 5'-extension to exon 3. It should be noted that the transcriptional orientation of the provirus in tumor 1206 was opposite to that of *Bach2*, thus minimizing the possibility that the virus directly contributed such an alternative promoter.

In a first attempt to address the nature of a possible alternative *Bach2 *promoter we searched for indicative ESTs. One spliced EST sequence, AK042574, was identified which could have origin in the usage of a *Bach2 *intron 2 located promoter. By RT-PCR analysis we verified the existence of the RNA corresponding to the EST (data not shown). The first exon of the EST was denoted exon 2A. By usage of different primer combinations in various RT-PCR analyses, splicing from exon 2A to exon 3 was identified. However, no up-regulation of this transcript was observed in material from tumor 1206 compared to other tumors and normal spleen (Figure 2E). Thus, the promoter sequence in front of exon 2A seems not to be the target for the observed *Bach2 *deregulation in tumor 1206.

**Figure 3:**
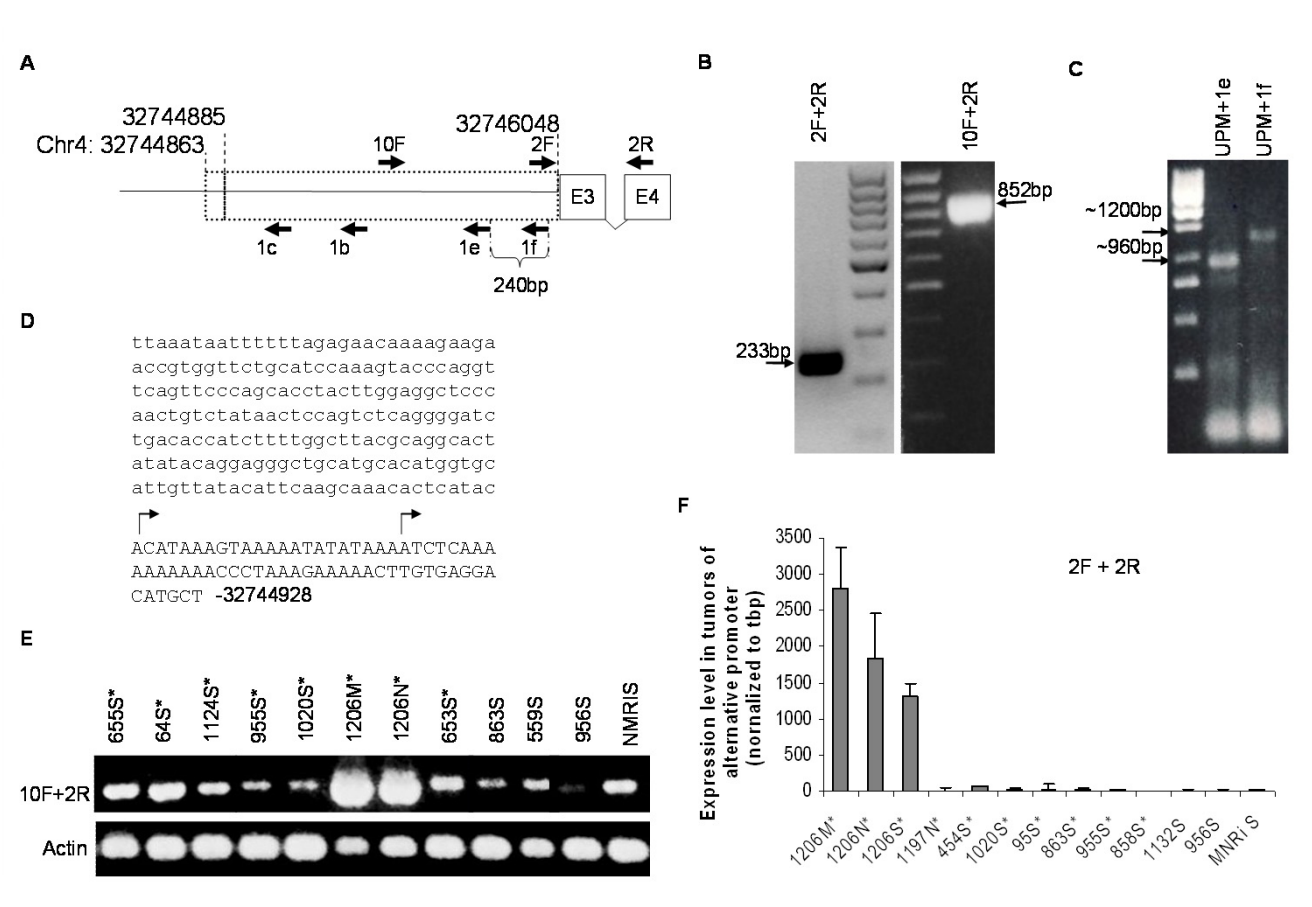
**Identification and characterization of an alternative *Bach2 *promoter**. (A) Map of the 5' ends for the novel mRNAs determined by 5'RACE and primers employed in 5' RACE PCR and sequencing (1c, 1b, 1e, 1f), and in RT-PCR and Q-PCR (10F and 2R and/or 2F and 2R). Chromosomal locations refer to UCSC version 2006 February. (B) RT-PCR detection of transcripts including intron 2 sequences as a novel *Bach2 *exon. Tumor material from mouse 1206 was analyzed by RT-PCR using primers 2F and 2R (left panel) or 10F and 2R (right panel) and by ethdium bromide staining. (C) 5'-RACE analysis to detect novel *Bach2 *promoters. The products of the RACE PCR analysis were visualized by ethdium bromide staining. Arrows indicate the product bands. (D) Sequence around the alternative promoter. Exon sequences are in capital letters and position of mapped transcriptional start sites indicated by arrows. (E) RT-expression analysis of transcripts derived from the alternative *Bach2 *promoter. Tumor material with (marked by asterisk) or without *Bach2 *gene proviral integration were analyzed by RT-PCR with primers 10F and 2R and the products visualized by ethdium bromide staining. (F) Q-PCR analyses of transcripts with origin from the alternative *Bach2 *promoter. Analyses were done using primers 2F and 2R on tumors with (asterisked) or without proviral integration in *Bach2 *and spleen tissues from untreated NMRI mice (NMRI S). Expression levels were normalized to tbp and NMRI S with the expression level for NMRI S set to 20.

In a further search for alternative promoter sequences within intron 2, the possibility of a continuous 5'-extension of exon 3 was examined. By RT-PCR analysis using mesenteric lymphoma RNA from mouse 1206 we could by the use of a reverse primer located within exon 4 and a forward primer located immediately upstream of exon 3 (primer 2F) (see Figure 3A for primer localizations) detect a band corresponding to a transcript including *Bach2 *intron2 sequences as a novel exon (Figure 3B, left). This was further substantiated by using a forward primer, 10F, located 667 bp upstream of exon 3 which also resulted in a PCR product corresponding to a *Bach2 *mRNA species including intron 2 sequences (Figure 3B, right).

In order to determine the 5' end of this novel mRNA subtype, we performed 5' RACE analysis on the mesenteric lymphoma RNA from mouse 1206 using either reverse primer 1e or 1f located upstream from exon 3 (see Figure 3A). Two distinct 5' ends of mRNAs were identified, located 1185 bp and 1163 bp upstream of exon 3, respectively (Figure 3C). We have found no evidence of the existence of splice donor sequences in the region between the alternative promoter sequences and exon 3, suggesting that the resulting transcripts indeed included a continuous 5'-extension to exon 3. The corresponding novel alternative first *Bach2 *exon was denoted E3L. No consensus TATA-box was present within the proximal promoter sequence. As translation of *Bach2 *initiates in exon 4, inclusion of E3L does not affect the *Bach2 *coding region. Moreover, no additional open reading frames were detected within the E3L sequences.

To examine if the alternative *Bach2 *promoter was active only in tumor 1206 material, we screened RNA from several tumors for the presence of such an alternative *Bach2 *transcript. By RT-PCR using forward primer 10F in combination with primer 2R we identified a 852 bp band in all examined tumors and in normal spleen corresponding to the Bach2 transcript including exon E3L (Figure 3E). Thus, the alternative promoter appears active both in tumor tissue and in normal spleen. Albeit semi-quantitative, the assay also pointed at an up-regulation of alternative *Bach2 *promoter in tumor 1206 as compared to the other examined tissue samples.

To further analyze the expression level from the alternative *Bach2 *promoter, Q-PCR assay was done using primers 2F and 2R (Figure 3F). This assay showed a 134-fold higher expression level of the *Bach2 *transcript resulting from the alternative promoter within mesenteric lymphoma from mouse 1206, when compared to the average expression level in the other examined tumors and in normal spleen (Figure 3F). Thus, the alternative *Bach2 *promoter was highly activated in tumor 1206. The amount of *Bach2 *transcripts derived from the alternative promoter was similar in all types of tumor tissues examined from mouse 1206 (Figure 3F).

### Activity comparison between the alternative and normal Bach2 promoters

**Figure 4:**
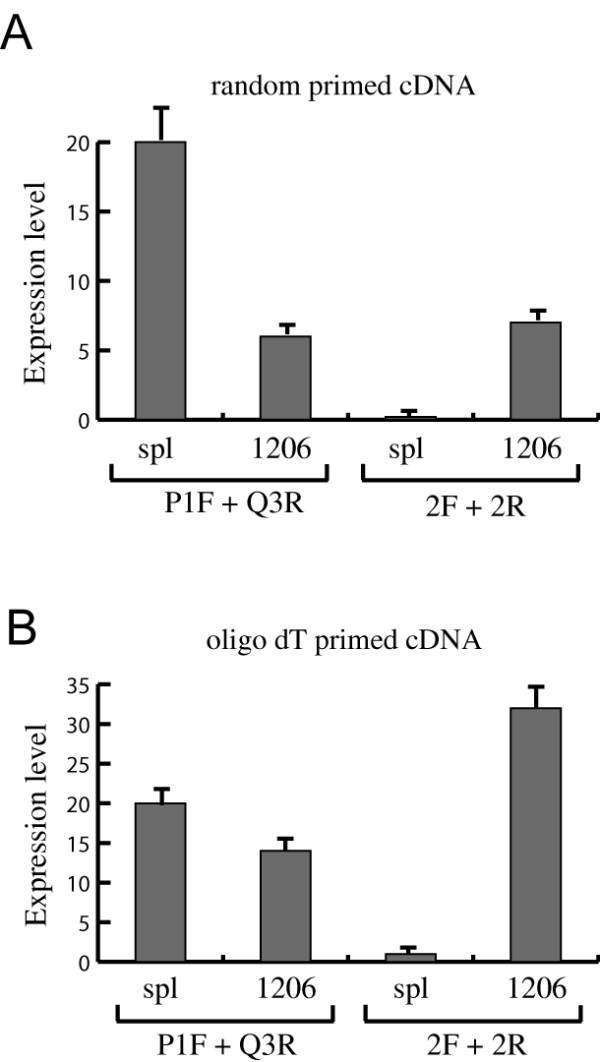
**Expression level analysis of *Bach2 *mRNA derived from the normal or alternative promoter**. Quantitative RT-PCR analyses were performed with primer pairs amplifying either cDNA representing transcripts derived from the normal *Bach2 *promoter (primers P1F and Q3R) or cDNA representing transcripts derived from the alternative promoter (primers 2F and 2R). RNA was extracted from NMRI mice spleen (Spl) or tumor 1206 material and cDNA generated using either random-primed first strand synthesis (panel A) or oligo dT primed first strand synthesis (panel B). Expression levels were normalized to the tbp expression level and the expression levels for the normal Bach2 transcript in NMRI spl set to 20.

To examine the relative amount of *Bach2 *transcripts derived from the alternative promoter compared to the normal promoter we used a Q-PCR based assay. cDNA representing the *Bach2 *transcript derived from the normal promoter was amplified using primer pairs in exon 1 (primer P1F) and exon 3 (primer Q3R) and cDNA representing the *Bach2 *transcript from the alternative promoter was amplified using a primer located in exon 3L immediately upstream of the beginning of exon 3 (primer 2F) and an exon 4 primer (primer 2R). The amplification efficiency for the two *Bach2 *amplicons was determined to be equivalent (~95%) as estimated from Q-PCR reactions performed on serial dilutions on purified cDNA representing the two *Bach2 *transcripts (data not shown). Q-PCR analyses were performed on cDNA from tumor 1206 and from normal spleen and both random primed and oligo dT primed cDNA was examined to account for bias in cDNA synthesis reactions (Figure 4). In cDNA synthesised from tumor 1206 mRNA the amount of the two types of *Bach2 *cDNA was comparable (Figure 4). This similarity in expression levels was observed both in random-primed and oligo dT primed cDNA (Figure 4A and 4B). In normal spleen the level of the *Bach2 *cDNA derived from the normal promoter was comparable with the cDNA level in tumor 1206 whereas the amount of the alternative promoter derived *Bach2 *cDNA was present in a low amount (Figure 4).

**Figure 5:**
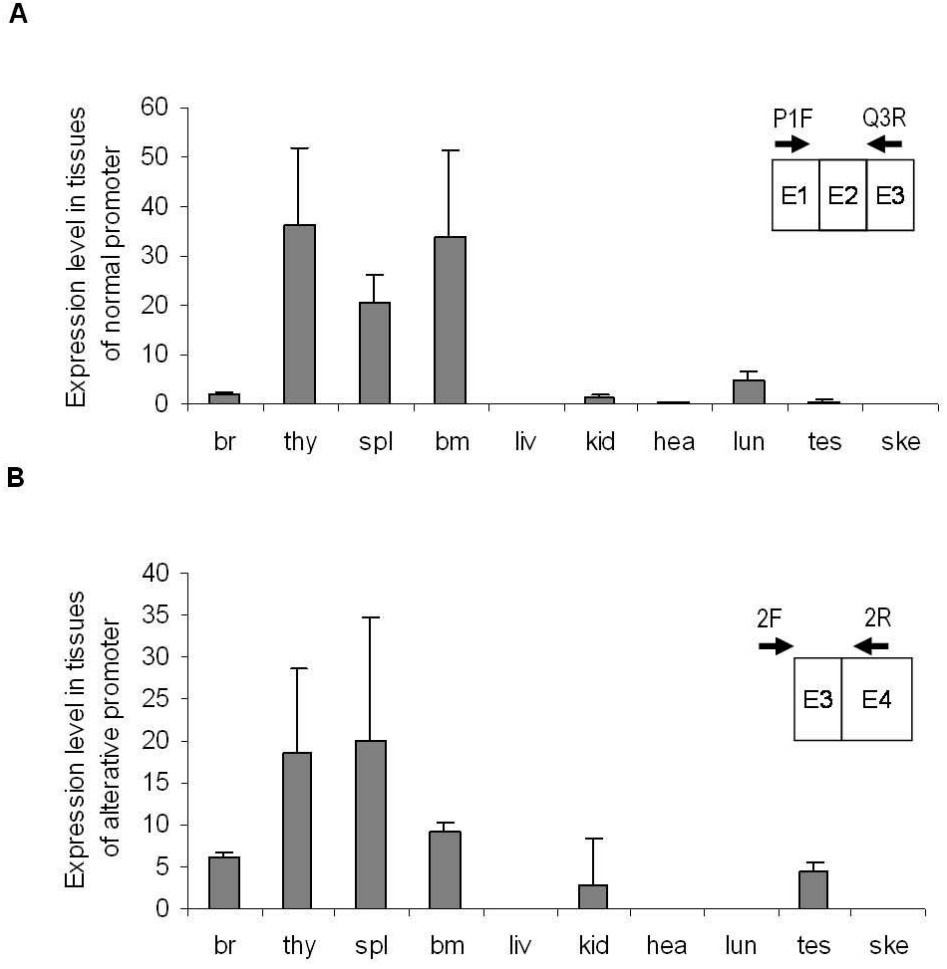
**Expression in mouse tissues of *Bach2 *mRNA derived from the normal or alternative promoter**. Q-PCR analyses were performed with primer pairs amplifying either transcripts derived specifically from the normal *Bach2 *promoter (panel A) or transcripts derived from the alternative promoter (panel B). cDNAs used for the assay were obtained from NMRI mice not infected with retrovirus. br, brain; thy, thymus; spl, spleen; bm, bone marrow; liv, liver; kid, kidney; hea, heart; lun, lung; tes, testis; ske, skeletal muscle. Expression levels were normalized to total RNA and spleen tissue; expression level for spleen by normal promoter was set to 20.

We next examined the tissue specificity of the alternative *Bach2 *promoter relative to that of the normal promoter. RNA representing ten types of organs from mice not infected with retrovirus was examined by Q-PCR using primer pairs amplifying sequences representing the two *Bach2 *transcripts as described above. As seen in Figure 5, transcripts originating from both the alternative and the normal promoters were more abundant in hematopoietic tissues, for example thymus, spleen, and bone marrow, compared with that in other tissues such as kidney, heart, lung, or skeleton muscles. Interestingly, in brain and testis, the alternative promoter tends to be utilized relatively more than the normal promoter as compared to the hematopoietic tissues (Figure 5). We note that in none of the examined tissues the alternative *Bach2 *promoter seemed to be the major source of Bach2 transcripts.

### Alternative usage of Bach2 terminal exons

The identification of the alternative *Bach2 *mRNA isoforms described above points to a transcriptional complexity of the *Bach2 *locus. To address this further we by *in silico *analysis searched for EST sequences representing other alternative *Bach2 *exons. Several such putative exons were identified and illustrated in Figure 5. The ESTs BC099420 and AK162095 are indicative of the presence of two novel alternative *Bach2 *exons spliced to the exon 4. These two exons are denoted exon 5A and exon 5B. Exon 5A (second exon in BC099420, the first being exon 4) contains a consensus poly-A signal, and the corresponding EST sequence includes a poly-A tail. Thus, the inclusion of exon 5A in the *Bach2 *transcript appears to generate a transcript in which exon 5 to exon 8 are skipped. The existence of *Bach2 *mRNAs containing exon 5A was verified by RT-PCR and sequencing of tumor material from mouse 1206 (data not shown). Also the existence of a spliced transcript including exon 3, exon 4, and exon 5A was identified in tumor 1206 material (data not shown). We have not been able to detect *Bach2 *mRNA containing exon 5A in any tumor samples except 1206, or in normal spleen tissue (data not shown). A stop codon is present in exon 5A in frame with the *Bach2 *start codon located in exon 4.

The existence of *Bach2 *mRNAs including exon 5B (the second exon of EST AK162095) was verified by RT-PCR and sequencing of tumor material from mouse 1206 (data not shown). Again, we were not able to detect mRNA containing exon 5B in other tumor samples than tumor 1206, or in normal spleen tissue (data not shown). The 3'-end of the exon 5B sequence derived from EST AK162095 includes no poly-A signal, and therefore might be protruded to the polyadenylation signal located for exon 4A. We note a distance of 15 kb between the poly-A site of exon 5A and the 3'-end of the EST sequence of exon 5B, and accordingly we have not been able to determine if exon 5B indeed is extended to the same polyadenylation signal as exon 5A (data not shown). We also note that we have been unable to detect splicing between exon 5B and downstream *Bach2 *exons by RT-PCR. A stop codon is present in exon 5B in frame with the *Bach2 *start codon located in exon 4. According to the rules of non-sense mediated decay splicing of exon 5B to exon 5 will generate a substrate for degradation supporting that exon 5B in itself contains a polyadenylation signal.

From the RT-PCR analyses, it was evident that still another alternative exon sequence exists. This exon, denoted exon 4 L, results from the absence of splicing between exon 4 and exon 5B and accordingly have a termination similar to exon 5B (data not shown). *Bach2 *transcripts including this exon 4 L was detected in tumor 1206 material as well as in other tumor samples, and also in normal mouse spleen (data not shown). Splicing between exons 3 L and 4 L, as well as between exons 1, 2, 3, and 4 L was detected by RT-PCR, and thus both the normal and the here identified alternative *Bach2 *promoter may generate *Bach2 *transcripts including exon 4 L.

### Inclusion of alternative Bach2 terminal exons results in generation of novel Bach2 protein isoforms

**Figure 6:**
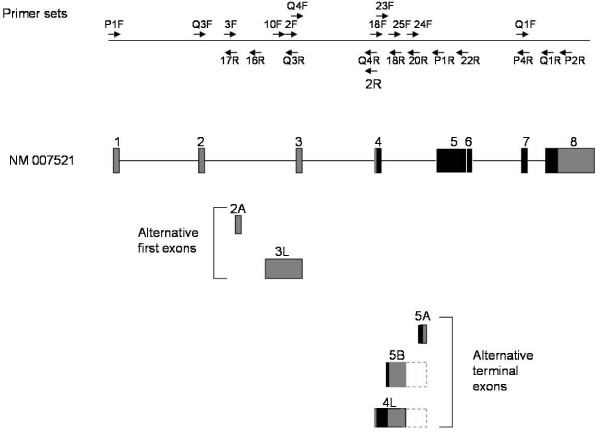
**Genomic structure for *Bach2 *gene with positioning of alternative exon sequences**. Top panel depicts primer sets used in the identification of *Bach2 *mRNA isoforms. Coding sequences were shown in dark boxes and non-coding sequences in grey. For exon 5B and 4 L the 3'-end of the exon was not mapped and the possible extension to the polyadenylation signal present in exon 5A was indicated by a dashed box.

**Figure 7:**
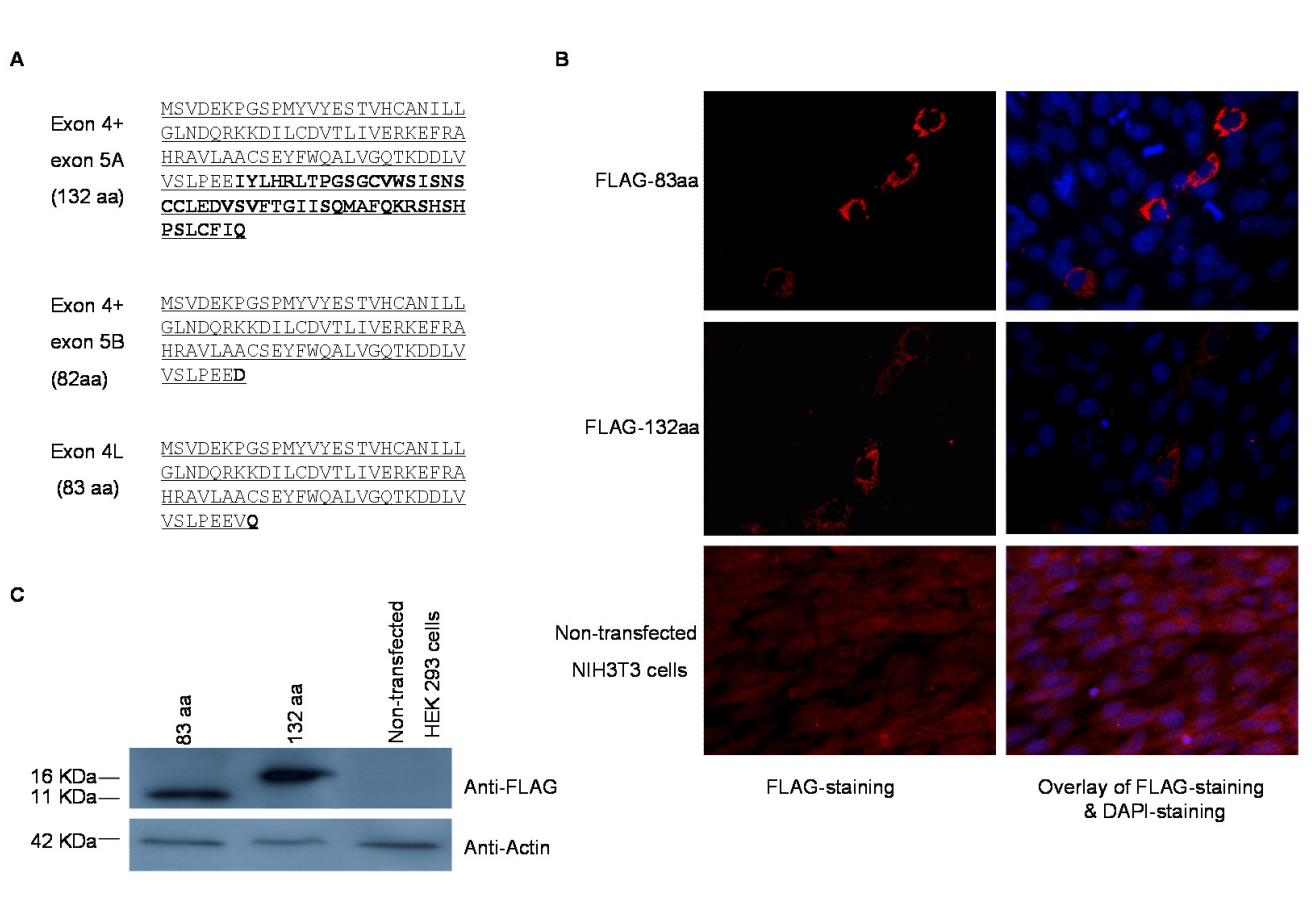
**Identification of novel Bach2 protein isoforms**. (A) Amino acid sequence of the Bach2 protein isoforms resulting from translation of *Bach2 *mRNA including exon 5A, 5B, or 4 L. BTB domain sequences are underlined and the sequences different from the canonical BTB domain are in bold letters. (B) Sub-cellular localization of the Bach2 132 aa (encoded by exon 5A including transcript) and 83 aa (encoded by exon 4 L including transcript) isoforms. NIH-3T3 cells were transfected with expression vectors encoding N-terminal FLAG-tagged open reading frames of the Bach2 isoforms. 48 hours after transfection cells were immunostained with anti-FLAG antibody and a TRITC-conjugated secondary antibody. Fluorescence was monitored by epi-fluorescence microscopy. DAPI staining was used to localize the nuclei. Non-transfected NIH3T3 cells served as negative control. (C) Western blot analysis of the Bach2 proteins. HEK-293 cells were transfected with the FLAG-tagged Bach2 expression constructs described in (B) and cellular extracts analyzed by western blotting using an anti-FLAG antibody. Molecular weight markers are indicated to the left.

The inclusion of exon 5A, 5B, or 4 L in the Bach2 transcript results in the generation of C-terminally truncated *Bach2 *ORFs, which lack the b-ZIP DNA binding domain. The coding regions in common share the N-terminal part of the Bach2 BTB domain encoded by exon 4. This segment constitutes the major part of the BTB domain (see Figures 1 and 6). Inclusion of exon 5A or exon 5B in the *Bach2 *transcript results in the generation of 132 and 82 amino acids *Bach2 *ORFs, respectively, in which the first 81 amino acids are identical. Inclusion of exon 4 L results in the generation of a 83 amino acids ORF in which the first 81 amino acids are identical to the Bach2 isoform encoded from exon 5B (Figure 7A).

The canonical Bach2 protein is localized in the cytoplasm by its C-terminal cytoplasmic localization signal and nuclear-export signal [7]. To determine the localization of the alternative Bach2 protein isoforms, we cloned the ORFs of the 132 and 83 amino acid variants in the mammalian pSG5FLAG expression vector, which accordingly was used to express the Bach2 proteins in NIH 3T3 cells or HEK 293 T cells as FLAG tagged fusion proteins. Immunofluorescence analysis showed that both proteins were nuclear-excluded (Figure 7B). Instead a peri-nuclear localization was observed (Figure 7B). No difference in the localization of the 132 amino acid and 83 amino acid Bach2 protein isoforms was evident. Thus, the addition of the C-terminal extension in the 132 aa Bach2 isoform had no clear consequence on localization. The integrity of the expressed Bach2 proteins was confirmed by Western blotting (Figure 7C). In conclusion, transcription from the *Bach2 *locus directs expression of alternative protein isoforms with the same subcellular localization but different composition of functional domains.

## Discussion

We here report proviral integration into the *Bach2 *gene in 18 independent B-cell lineage tumors induced by Akv or Akv derived MLV in NMRI mice. Proviral integration was identified and confirmed by PCR analysis and sequencing. All cases of proviral insertion were located in intron sequences upstream of the translational start codon of the canonical *Bach2 *mRNA or within the *Bach2 *promoter region and most of the integrations were in the opposite transcriptional orientation to that of the *Bach2 *gene. Thus, our analyses support previous findings of *Bach2 *locus being a common integration site (CIS). Fourteen *Bach2 *integrations are reported in the Retrovirus Tagged Cancer Gene Database (RTCGD) [32,33], thirteen of which originate from Akv induced malignancies in mouse strains of AKxD and NFS [34,35]. The distribution pattern of these 13 integrations shows remarkable similarity to what we have demonstrated in the present study: they all are B-cell-related, located in non-coding sequences from the promoter region to intron 3, and predominantly having an inverse orientation. The frequency of targeting specific genes depends on both the mouse host strain and the type of retrovirus. In one mouse strain different types of malignancy can be induced by different retroviruses. For example, in NMRI mice Akv and SL3-3 MLVs induce B- and T-cell lymphomas, respectively [36-38]. The same retrovirus may also behave diversely in various host strains. For example, the *Icsbp *gene is an Akv-related target in NMRI mice, but not in other mouse strains such as AKR, AKxD, and NFS [39-42]. *Bach2 *seems to be a target gene in a variety of host genetic backgrounds for Akv induced B-cell malignancies. Besides the thirteen Akv MLV integration sites, RTCGD contains one Moloney MLV integration site within in the *Bach2 *gene, which is derived from a brain tumor in the mutated C57BL/6 (Ink4a/Arf (-/-)) strain [43]. This integration site is located at intron 4, and accordingly disrupting the coding sequences. Notably, in humans, BACH2 was also identified to be recurrently integrated by HIV in human CD4^+ ^T cells [44]. These integrations were all in intron 5 and had all same transcriptional orientation as the BACH2 gene.

By Southern blot analysis using an ecotropic envelope probe and *Bach2 *gene specific probes, we analyzed clonality of the B-lymphomagenic tumors induced by Akv and Akv derived MLV in inbred NMRI mice. By this approach we have not been able to detect the virus integration within the tumor in accordance with only a small fraction of the tumor cells having the actual integration. Such a Southern blot pattern resembles what we have described in a previous report for Akv MLV derivates in the NMRI mouse strain [23,41]. A model for this observation could be that B-cell lymphoma induction by Akv MLV in the inbred NMRI mouse strain may involve immune stimulation [24]. Such stimulation may cause an initial polyclonal stimulation followed by multiple events of mutagenesis by proviral insertion. Given that only a minor fraction of the tumor cells harbours the proviral integration, it may not be surprising that in most cases we were unable to detect *Bach2 *transcriptional deregulation. Still, in tumors from mouse 1206 transcriptional upregulation of *Bach2 *was detected. This upregulation was however only observed for exon 3 to exon 8 sequences, and accordingly pointed to activation of an alternative *Bach2 *promoter located in intron 2. Indeed, we were able to identify such a novel Bach2 promoter and in accordance with the expression data we observed increased expression within tumor material from mouse 1206. Moreover, we found that in tumor 1206 material the expression level of the *Bach2 *transcript derived from the alternative promoter was similar to the expression level the *Bach2 *transcript driven from the normal promoter.

Interestingly, the alternative *Bach2 *promoter was also identified to be active in normal mouse tissues and accordingly could play a role in the normal regulation of *Bach2 *expression. The tissue specificity of the two *Bach2 *promoters was overlapping, but we note that the alternative promoter in some non-hematopoietic tissues had an increased relative expression indicating that the contribution to the overall *Bach2 *mRNA level could be of physiological importance in such tissues. We did not observe normal tissues in which the alternative promoter derived *Bach2 *transcript was expressed at a higher level than the normal *Bach2 *transcript, but the results point out that transcription directed from this alternative *Bach2 *promoter is important to include in future expression analysis addressing *Bach2*. Usage of the intron 2 promoter will if the transcript is spliced from exon 3 L to exons 4, 5, 6, 7 and 8 result in generation of the normal Bach2 protein, as the start codon is located in exon 4. The identification of proviral integrations also in the normal promoter region of *Bach2 *and in intron 1 supports that upregulation of the canonical Bach2 protein is involved in MLV-induced tumorgenesis.

Our RT-PCR analysis showed that besides the usage of alternative promoter sequences *Bach2 *gene regulation also involves alternative usage of terminal exons. Such terminal exons were identified in intron 4 of the *Bach2 *gene. The inclusion of alternative terminal exons resulted in the skipping of exons 5 to 8 from the *Bach2 *transcript. Transcripts arising from both the normal *Bach2 *promoter and from the novel *Bach2 *promoter were identified to include such alternative terminal exons and the resulting transcripts have coding potential for novel subtypes of Bach2 protein having C-terminal truncations and inclusion of new amino acid compositions. The canonical Bach2 protein consists of both a BTB domain at the N-terminus and a bZip domain near the C-terminus. All of the deduced proteins for these isoforms possess a fragment of the BTB domain but lack the bZip domain. The BTB domain, also known as the POZ (poxvirus zinc finger) domain, was originally identified as a conserved motif in the Drosophila proteins bric à brac, tramtrack and Broad-Complex [45,46]. The BTB domain is involved in a variety of cellular functions, including transcription repression [47], cytoskeleton regulation [48,49], and targeting protein for ubiquitination/degradation [50-53]. The BTB domains from the BTB-bZip proteins (Bach1 and Bach2) are of an elongated form (around 120 residues in total), containing an additional amino-terminal region to the highly conserved BTB core region which consists of 95 residues [54]. As protein-protein interaction domains, the BTB domains are involved in forming homo- and hetero-dimerization, as well as protein oligomerization, with a specificity depending on their amino-acid sequences and structure (reviewed in [3]). Note the BTB domain was shown not to be required for the interaction between Bach2 and B-cell lymphoma 6 (BCL6) proteins *in vitro *[55]. Although the BTB sequences included in the hereby identified novel short Bach2 protein isoforms are truncated compared to the BTB domain in normal Bach2, they share the amino-terminal region which was predicted to confer stable dimerization [54]. We propose that these alternative proteins may play roles distinct to normal Bach2 either independently or by forming competitive protein interactions in for example the B-cell compartment with normal Bach2 protein. The involvement of such small Bach2 proteins in tumorgenesis is also an interesting possibility as they could have a dominant negative effect on normal Bach2 functions through formation of protein-protein interactions but generating protein complexes that lack the DNA binding domain normally contributed by Bach2. In light of the fact that Bach2 is not expressed in the plasma cell stage, Bach2 proteins most probably play their part in upstream steps of the tumorgenesis. Functional analyses of alternative Bach2 transcripts and proteins are in progress to further elucidate this issue and clarify the importance for normal cellular regulation as well as B-cell lymphomagenesis.

## Conclusion

The common insertion of proviruses at the Bach2 locus in a murine model of B-lymphomagenesis provides strong evidence that mutation at this locus plays a role in the disease. In this work we have identified an alternative promoter and new protein isoforms of Bach2 and our data imply that activation of an alternative promoter by proviral integration serves as a possible mechanism of up-regulation of the *Bach2 *gene with a potential role in B-cell lymphomagenesis. Such differential expression of protein isoforms with distinct functions may explain why the *Bach2 *gene, previously suggested to be a tumor suppressor may be up-regulated in B-cell lymphomas. The finding of novel *Bach2 *transcripts and protein isoforms will facilitate a better insight into the normal and pathophysiological regulation of the *Bach2 *gene.

## Authors' contributions

JL carried out all experimental work except large scale screening for proviral insertion sites and the data presented in Figure 4 and wrote the first manuscript draft. BW and MW performed the large scale screening for proviral insertion sites. ALN performed the Q-PCR analysis presented in Figure 4. ABS, ALN, and FSP conceived of the study and contributed to the design and interpretation of experiments as well as to editing of the manuscript.
